# Revitalizing health for all: Case studies of the struggle for comprehensive primary health care

**Published:** 2017-12-15

**Authors:** Lori Hanson

**Affiliations:** 1 Department of Community Health and Epidemiology, University of Saskatchewan, Saskatchewan, Canada

2018 will mark the fortieth anniversary of the Alma Ata Declaration on Primary Health Care.^1^ That historic occasion is all the more reason to take note of the recent release of *Revitalizing Health for All: Case studies of the struggle for primary health care,*^2^ a new compilation of global experiences and research into the 40-year old ideal of “Health for All through Comprehensive Primary Health Care” or CPHC. Exceedingly rare are such global collections – making this book a valuable addition to teaching the varied applied ways in which the concept of primary health care has been launched, interpreted, morphed and implemented in policy and practice around the world.

*Comprehensive primary health care* is a sociopolitical concept and philosophy of health care that must be differentiated from *primary care* as we frequently utilize that concept in Canada to indicate the first level of contact with health services. Yet few students are introduced, much less versed, in the nuanced ways in which the terms differ and are interchanged. As the editors of this book note, the original intent of CPHC was to be a “sociopolitical philosophy as well as an implementation strategy for improved health equity” through access to health care and the social determinants of health, made possible by political and technological choices. CPHC in the Alma Ata Declaration embodied five essential principles: universal accessibility and coverage based on need; comprehensive care; inter-sectoral collaboration and action on social determinants; active community participation; and appropriate technology. Putting those principles in practice inevitably led to changes, challenges, and appropriations – which are succinctly laid bare in the introductory section of the book.

Taken from a Global Health Fund Initiatives research-to-action project carried out from 2007–2011, and with publication support from the International Development and Research Centre, the initiatives documented include 50 collaborators and 13 cases from four continents – largely but not exclusively in low and middle income countries. In the book, the initiatives are categorized into four thematic sections reflecting CPHC’s comprehensive nature: 1) increasing equitable access to health care, 2) community engagement, 3) community health workers, and 4) governance and inter-sectoral action. Each section is briefly introduced, and overall the collection is flanked by introductory and concluding commentary by the editors. With a purpose that is not entirely clear, the four introductory sections are sprinkled with brief synopses of research projects from teams that hadn’t submitted full chapters – some in text boxes and others described in the narrative.

The editors are an experienced team. Dr. Labonte, from the University of Ottawa, is currently Canada’s Research Chair in globalization and health equity, a position he came to after a long history of work in health promotion. Dr. Sanders, a pediatrician and public health specialist from the University of the Western Cape, South Africa, is a world-renowned advocate and author on comprehensive primary health care. Corinne Packer, as a senior University of Ottawa researcher, has written on CPHC, and Nikki Schaay teaches modules on CPHC at the University of the Western Cape.

After a background on the research project’s rollout, the book commences its topical discussion with a literature review, highlighting strengths and weaknesses of the evidence on CPHC to date and organizing the findings by region. Some of the cited limitations of the published literature on CPHC include the paucity of national studies of CPHC experiences; preponderance of descriptive and evaluation studies; over-emphasis on behavioural aspects of change over social determinants of health; and emphases on particular pieces of a program, rather than a comprehensive account of the whole. Integrating findings from the grey and scientific literature, the evidence also suggests that CPHC remains rooted in improving access to health systems for marginalized groups, and that both community governance and politics matter in sustaining funding and support for CPHC. Conversely, they write, a “focus on hospital care and the dominance of the biomedical model of health constrained support for CPHC.” (p. 43)

As was the global research project, the book is ambitious, with the cases in it reflecting the diversity of experiences and front line struggles that the title alludes to, with correspondingly varied results. Notably, CPHC as envisioned in Alma Ata has nowhere been *fully* instituted as a national health system by national governments working in concert with communities, nor has it been awarded time and sufficient resources to develop as conceived.

Consequently, studies with comprehensive evaluations of CPHC as a national or regional system are rare. Had they existed, books on the topic would be multiple and country-level comparisons relatively straightforward. Instead, there have been diverse country, region, community-level and nongovernmental organization experiments in CPHC that represented important, but typically partial, time-bound or incomplete translations of the idea. As the book points out, research evidence of the effectiveness of CPHC is thus equally piecemeal.

Little wonder then, that the editors make no apology for the variance in quality of the studies reported on in the book. Nonetheless, for the medical educator wishing to utilize this collection in its entirety or to select exemplary cases for illustrating practices and problems related to CPHC, the unevenness can be challenging, and requires careful review. Suggested for optimal use would be presentation of a smattering of the cases, giving each due introduction and explanation of the variance with which CPHC has been historically interpreted in practice. Discussion could follow, concentrating on how interpretations of health equity in CPHC practice varies across different country and local contexts, being enabled or restricted by access to resources, by levels of community organization, by macro-political trends, and by political will. In that way, the diversity of presentation of the cases would succeed in highlighting the challenges of translation of CPHC from principles to action in real-world, often low resource contexts.

The book’s first section, *Increasing equitable access to health care* showcases experiences from specific programs in Australia and the Democratic Republic of Congo, and describes the process of implementation of CPHC in the city of Bogota, Colombia. The case of Alice Springs’ Male Health Program illustrates the importance of building responsiveness and flexibility into a program, principles that allowed the team to re-think strategies when problems were encountered, and thereby increase accessibility and coverage of services. The case of Colombia suggests tensions inherent in combining models of CPHC and primary care, and suggests those as consequences of fragmented health systems.

The second section of the book relates to the core principle of community participation (CP), curiously using the more modern nomenclature: *Community Engagement*. In this section, the research documented comes from South Africa, Kenya, and Bangladesh. Set up as a cross case study of community health worker involvements with communities, the research from South Africa suggests that successful community engagements – those that affect equity – both need and need to support networks of social capital within communities. Such support implies work beyond the realm and even the reach of the health system. The reports on the mixed methods research on initiatives in Kenya and Bangladesh are framed within historic system-level inadequacies and shortcomings vis-à-vis community involvement. Thus the rather sobering introductions valuably situate the study’s findings - far short of the ideals of CP - in context. Although serious constraints are evidenced, the trends visible in both studies confirm the premise that community involvement is vital to attaining improvements in health outcomes through CPHC.

The third section contains accounts that make more apparent the links among health systems, health outcomes and community interventions. Herein, the CPHC cornerstone - *Community Health Workers* - is explored through diverse experiences from Iran, India, and Ethiopia. Ethiopian Health Extension Workers’ experiences are so vast and multiple that two cases are included. Although some incomprehensibility occurs due to the complexity of study measures and performance indicators, the studies taken together present compelling pictures of the essential function that community health workers play in enacting CPHC goals.

The complexities of implementation of CPHC-based systems across levels of care comprise the substance of the fourth section of the book – *Governance and Intersectoral Action.* Here within-system comparisons are drawn from national implementation experiences, including Brazil’s Family Health Strategy and Argentina’s primary health care framework as enacted in two sites. The section ends with what is perhaps the best case scenario – and clearly an editorial favourite – from El Salvador’s community-driven CPHC experience in Guarjila.

Overall the collection presents a compelling if problematic picture. As the editors clearly note, well-designed and well-funded *research* on CPHC remains scarce. If systems and communities are struggling to implement even small projects adhering to CPHC principles, even less resources are allotted to its measurement and to meaning-making – the stuff of research. While the project and this publication have made an honorable attempt at addressing this shortfall, the editors neglected an opportunity to summarize lessons learned about *methodological* issues highlighted by the processes.

Still, there is much to be learned and good teaching material here.

Sometimes subtle, sometimes loud, the projects cited in this collection are all peppered with political and economic undertones. Some authors are kinder than others to the national regimes in which their experiences have unfolded. But most reveal how historic political choices and current neoliberal ideologies have worked against the implementation of CPHC thereby giving rise to health system issues such as vertical programs and fragmentation, and justifying the deepening of inequalities through programs of austerity. Against that backdrop, it would seem a distant possibility that CPHC might have a future. And yet that is precisely what the book presents – the live, tangible, current possibilities within struggles for CPHC. Fitting then, that it ends on a note of “cautious optimism.” In answer to the final chapter’s title: *Conclusion: Is there a future for Comprehensive Primary Health Care?* the editors suggest that the answer is “yes,” but with the caveat that political commitment to health equity is requisite. Still, they conclude, “it is an idea too powerful to disappear.”
